# Dental trainee motivations for the acquisition and use of postgraduate qualifications in medical education

**DOI:** 10.1038/s41415-024-7054-z

**Published:** 2024-02-09

**Authors:** Philip A. Atkin, Kiran K. Saini

**Affiliations:** Cardiff Dental Hospital and School, University Hospital of Wales, Heath Park, Cardiff, CF14 4XY, UK

## Abstract

**Introduction** Medical and dental education is increasingly professionalised. The expectation is that educators have appropriate training and qualifications. Entry to dental speciality training is highly competitive and applications are scored using defined criteria, including experience in education.

**Material and methods** We surveyed a group of junior trainees who had enrolled on postgraduate medical education courses to find out more about their motivations and use of their knowledge and qualifications. An online survey tool was used with anonymous participants who had been in junior training jobs in a university dental school, with 117 possible participants.

**Results** In total, 61% of respondents were enrolled in or had completed a postgraduate qualification in medical education, with 85% on a postgraduate certificate programme. Additionally, 77% were in the earliest part of general dental training, and 88% of programmes were distance-learning and cost £2,000 to £4,000. Motivations for enrolling included to ‘improve knowledge in medical education' and ‘increase my chances of progression through training'.

**Discussion and conclusions** We found the most common intrinsic reason to gain a qualification was to improve knowledge in medical education and the most common extrinsic reason was career progression. Scoring of applications into dental speciality training has changed, with points no longer awarded for completing a postgraduate qualification in medical education.

## Key points

- Demonstrates the appetite for gaining postgraduate qualifications.
- Explores motivation and use of postgraduate qualifications.
- Identifies costs of engaging in postgraduate training.

## Introduction

Professionalisation of medical education has been more than 100 years in the making. In relation to early formal medical education in the UK, the 1815 Apothecaries Act ‘provided for state control of practice and spelled out precise regulations for training, both theoretical and practical'. The Medical Act of 1858 created the General Council of Medical Education (now the General Medical Council) which had to ensure adequate standards of medical education.^1^ Transition from an apprenticeship model to a formal educational model, the study of medicine was described in a letter to *The Lancet* in 1860 which said ‘professional study is considered to commence with the first entrance and attendance at a recognised hospital or medical school. Apprenticeship or pupillage is not of itself professional study'.^2^ In 1944, the *Goodenough report* recommended major changes to UK undergraduate medical education with a ‘drastic overhaul of undergraduate training.'^3^ A similar review had been carried out in the USA in 1910: the *Flexner report* suggested major overhaul of US medical schools and the core curriculum they should all follow.^4^

The development of professional medical educators was to follow sometime later. In 1985, one of the first ‘training the trainers' courses was established in response to dissatisfaction expressed by specialist trainees in community medicine regarding the quality of training and education they were receiving.^5^ This was one of the early attempts to give trainers and educators the knowledge and skills necessary to effectively train junior colleagues. The 1997 *Dearing report* regarding all UK higher education (HE) establishments and their delivery of postgraduate education also recognised the need to ‘establish a professional institute for learning and teaching in higher education. The functions of the institute would be to accredit programmes of training for higher education teachers; to commission research and development in learning and teaching practices; and to stimulate innovation'.^6^The Institute of Learning and Teaching (now rebranded as Advance HE - formerly the Higher Education Academy) was able to accredit programmes of postgraduate education and register those who completed such courses as members.^7^ In medical education, the Academy of Medical Educators (AoME) was formed in 2006 to recognise and accredit those involved in the organisation and delivery of medical education.^8^ There are a large number of UK higher and further education institutions that offer formal qualifications in medical, dental or clinical education, which may then be used to support individuals' applications to bodies such as Advance HE or AoME, which then lends credibility to medical educators in the workplace.

On graduation, most newly qualified dentists enter a year of foundation dental training based in general dental practice and overseen by regional dental postgraduate dental deaneries. Some dentists may then progress into dental core training posts from between one (DCT1) and three (DCT3) years. Typically, the DCT1 posts are split between hospitals with dental specialties and maxillofacial surgery (dental hospitals or district general hospitals [DGHs]) and community dental services, with the aim of the trainee experiencing a number of dental specialties. DCT2 and DCT3 posts are more usually based in dental hospitals or DGHs and are more dental speciality focused. During this period, dentists will aften sit for postgraduate examinations which demonstrate the knowledge and competencies of a dentist with two years of training and experience, and any additional qualifications that may support applications into dental speciality training programmes (for example, dental education).

We recognised that within dental hospitals and schools, an increasing number of dentists in early-career training without formal responsibility for delivering educational activity were enrolling onto postgraduate programmes to gain qualifications in medical, dental, or clinical education and we were interested in finding out what the reasons for this were.

## Material and methods

Following ethical approval (Cardiff University DSREC 2028a), an anonymous online survey was conducted asking about the experience of undertaking a formal postgraduate qualification in medical education (including clinical or dental education) and the benefits or otherwise of completing such a qualification, particularly relating to the motivation for undertaking such a course and its subsequent use.

The pool of possible respondents was 117 current and former dental core trainees (DCTs) or senior house officers (SHOs) and speciality registrars (StRs) who worked in Cardiff Dental Hospital in the five years from 2017/18-2021/22. In the pool there were 22 StRs. DCTs and SHOs have typically completed one year of supervised dental foundation training in a family dental practice setting following dental qualification and speciality trainees have typically completed between 1-3 years as a DCT or SHO before entering speciality training.

Email invitations were sent explaining the survey and inviting participation. An online survey tool was used to create the survey and collect and help analyse the data.^9^ For validity, the questions used were based on questions from similar surveys on postgraduate medical education.^10^^,^^11^ Following the initial mail out, two reminders were sent out at one-week intervals before the survey closing to encourage a good response rate.^12^

Those invited to take part in the study were advised that there was no obligation to participate, that they could discontinue the study at any time, and that by completing the questionnaire they also consented to enter the study.

## Results

### Demographics

There were 54 respondents (46% response rate). A total of 33 (61%) respondents had completed or were undertaking a postgraduate qualification in medical education. Here, 89% (n = 48) of respondents were between 25-35 years old, which would match a group composed principally of DCT/SHOs and StRs. The male-to-female ratio was 1:3 and 96% (n = 52) had gained their primary dental qualification in the UK or Ireland - 72% (n = 39) of those within the recent five years. Of the group, 87% (n = 47) had gained the Membership Diploma of a Dental Faculty of a Royal College of Surgeons (MFDS), or equivalent. MFDS is an examination marker of knowledge and experience gained beyond two years of dental training and is undertaken by most DCTs/SHOs who plan to continue their careers into dental speciality training. Of the 26 respondents who had already completed a postgraduate qualification in medical education, 22 (85%) had undertaken a Postgraduate Certificate (PgCert), one a Postgraduate Diploma (PgDip) and three a Master's level degree. Many institutions will offer a rolling programme where interested participants can undertake a PgCert and then enrol into a second year for a PgDip and a third year for a Master's-level qualification.^13^

At the time of enrolment onto a teaching programme, 26 (77%) were in a DCT or SHO post, six (17%) were in non-training salaried posts and just two (6%) were already in StR posts. Of the 26 respondents who had completed a postgraduate qualification, 20 (77%) were in the early years (DCT or equivalent) of their career. Six of those who had already completed their postgraduate medical education course were members of the Higher Education Academy/Advance HE.

### Postgraduate courses

Participants identified their enrolment into postgraduate education courses from a wide geographical distribution of universities across the UK. Of the 31 respondents who identified their university of study, 21 (68%) undertook courses from just five institutions. Two-thirds of the courses were offered as distance-learning only, with smaller numbers for courses with study-day attendance. In total, 88% of the courses were offered for part-time study. Additionally, 79% of respondents identified the cost of their study as being between £2,000 (approximately €2,380/$2,618) and over £4,000 (approximately €4,720/$5,236). The course fees for the five most popular university courses for a one-year PgCert are shown in Table 1.

**Table 1:** University course fees for one-year postgraduate certificates in medical/dental education (as of March 2022)

Institution	Course	Fee
University of Bedfordshire	PgCert in Dental Education	£2,750
Swansea University	PgCert in Medical Education	£3,200
Newcastle University	PgCert in Dental Education	£3,400
University of Dundee	PgCert in Dental Education	£3,635
Cardiff University	PgCert in Medical Education	£4,850

## Discussion

### Motivation for enrolment on postgraduate medical education programmes

**Fig. 1:**
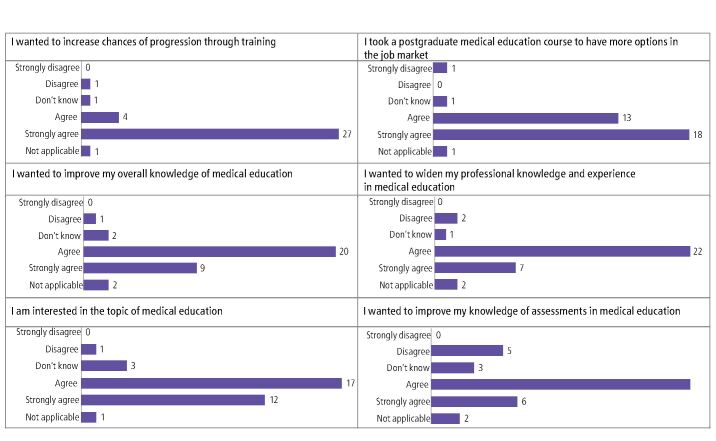
Motivation for enrolling on postgraduate medical education programmes

Figure 1 shows reasons of motivation to enrol onto postgraduate medical education programmes. In relation to a declared interest in the topic of medical education, 94% (n = 51) of respondents agreed/strongly agreed with the two statements ‘increase chances of progression through training' and ‘to have more options in the job market'. In relation to the statements ‘I wanted to improve my overall knowledge of medical education' and ‘I wanted to widen my professional knowledge and experience in medical education', 91% (n = 49) agreed/strongly agreed. Moreover, 85% (n = 46) of respondents agreed/strongly agreed they were interested in the topic of medical education and 75% (n = 40) agreed/strongly agreed that they wanted to improve their knowledge of assessments in medical education.

Box 1 shows some of the participant free-text responses in relation to motivation for enrolment on medical education programmes

Box 1 Free-text participant responses from participants regarding motivation for enrolling on postgraduate medical education programmes

- I enrolled in the [course] solely because this would help my StR application. However, this qualification has benefited me laterally as I am now in academia despite not aiming for this when undertaking the qualification. I am now keen to undertake another teaching qualification but in the process of determining which one
- The course was only demanding in the sense that it had to be completed in my free time (evenings/weekends)
- I knew I wanted to be an academic consultant, I pre-empted the possibility of needing a PGCert (or higher) in education, and/or the FHEA [Fellowship of the Higher Education Academy]. Additionally, I did them to improve my chances of obtaining academic specialist training
- Interested in the topic, maybe better to undertake at a different point in my career when I am doing more teaching
- My main reason to do the course is because it is needed for speciality training. I am disappointed it isn't a requirement anymore. It is really useful for tips for teaching and I think everyone should do this. Not everyone is a natural teacher/knows how to teach. This course opens you to ways of teaching dentistry. I think it also highlights the importance of being a great dental educator and is important. Sometimes I think it is a little early to do as a DCT2 as I am young with experience in dental teaching, but I can implement the tips going forward.

### Preparedness to use knowledge from postgraduate medical education programmes

**Fig. 2:**
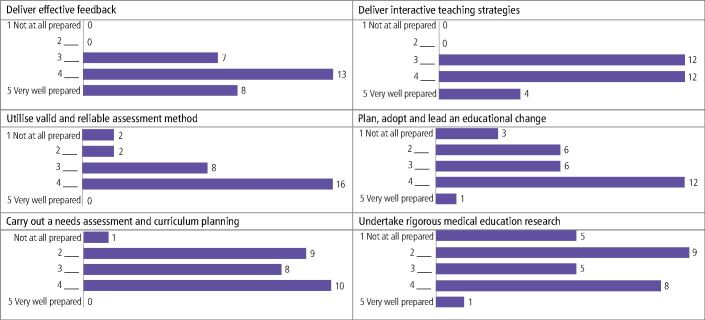
Preparedness to use knowledge from postgraduate medical education programmes

Figure 2 shows participant responses in relation to use of the knowledge gained from enrolling on a postgraduate medical education course. The large majority (75%, n = 40) felt they were prepared/very well prepared to ‘deliver effective feedback' and 57% (n = 31) felt prepared/very well prepared for both ‘delivery of interactive teaching strategies' and to ‘utilise valid and reliable assessment methods'. Just 44% (n = 24) felt prepared/very well prepared to ‘plan, adopt and lead an educational change'. Respondents were the least confident in ‘carrying out a needs assessment and curriculum planning' and ‘undertake rigorous medical education research' with only 36% (n = 20) and 32% (n = 17), respectively, feeling prepared/very well prepared for these areas.

Survey participants were asked if they would like to comment on any aspect of the survey, or their experiences of undertaking or completing a postgraduate programme in medical education. Their free-text responses are shown in Box 2.

The change in scoring applications for entry to speciality training, with the loss of points for being enrolled on or having completed a postgraduate course in dental education, is likely a response to the fact that most junior speciality trainees will do little teaching in their early years. Gaining knowledge and experience of teaching is better timed in the latter part of speciality training, when the next post will be as a hospital consultant who has trainees of their own to teach and train, or as a senior lecturer/honourary consultant in a dental hospital where there will be undergraduates as well as trainees to teach. This is where having a qualification in dental education will be most useful.

Box 2 Participant free-text responses in relation to their experience of, and motivation for, postgraduate programmes in medical education

- It was a very useful course and I use the strategies and teaching methods on a daily basis in both a formal and informal way
- My personal feeling is that DCTs feel they have to undertake this qualification purely for box ticking to increase scoring at national recruitment. Often this is at a stage in their career where they don't have clinical experience themselves and many may not have the opportunity to utilise their teaching skills regularly unless their DCT role involves supervision of undergraduates or other teaching opportunities. They may understand the benefits, but I suspect if it wasn't needed for national recruitment, many may delay this until later in their career? Perhaps too controversial?! However, undertaking the qualification at this stage of training likely ensures trainees can focus on it without too many day-to-day commitments. And the skills they gain are likely useful in their future career - I just suspect that might not be the primary reason for undertaking it at DCT level
- I found a postgraduate qualification in medical education very worthwhile as I had no prior teaching experience as a young dentist. I feel so many dentists now have at least the postgraduate certificate in medical education and as it is usually listed as a desirable criteria for StR jobs it would be of benefit for job applications. Within an StR role we have to undertake a teaching role daily. I felt it was important to understand the theoretical aspect of medical education and how to put that into practice if I was going to grow with confidence as a teacher
- The PgCert MedEd I am currently completing is more time consuming than initially anticipated. I am able to implement some of the teaching into my job role currently. I am disappointed to see it removed from the self-assessment questionnaire this year for StR posts
- My primary reason for doing the qualification was to get into speciality training
- I think questions on whether staff have sufficient time in their timetables and support from the [dental] school would be important to help indicate whether there's even the scope for staff to undertake these qualifications
- I think it is really useful. I think the cost of the course is an issue for some. I wish this course PgCert in medical education was highlighted in undergraduate studies. I think the many routes should be taught to us. I just randomly made the decision because of speciality [training] and everyone said I should do it! I didn't have all the info at hand.

### Findings

For this group of early-career trainees, enrolling on a PgCert in medical education matches their motivation to learn more about medical education in general, how to effectively deliver education, and how to consider appropriate modes of assessments for students in medicine and dentistry. However, their lack of experience of, or opportunities to become involved in, the delivery of medical/dental education means that application of that knowledge is limited. This is most likely because they are early in their careers and their training roles are not linked to undergraduate teaching and assessment. The primary motivation for enrolling in a PgCert course is to advance their career progression. This is reinforced by some of the statements in Box 1 and Box 2. At the time of the survey, the scoring matrix for application and shortlisting for interview for specialist training awarded points for being enrolled on a teaching qualification course and for completion of such a course. (Table 2). National recruitment into dental speciality training (StR) asks the applicants to fill in an application form with indicative points for each element. The application process is organised by Health Education England. The forms have a large generic element and smaller sections that are dental speciality specific. Examples of 2021^14^and 2022^15^ paediatric speciality training application forms show the points available for the different elements. In 2021, being enrolled on a PgCert in medical education scored as many points as possession of a Master's degree. Completion of a PgCert in medical education scored as many points as having a PhD. The cost and time needed to complete a PgCert in medical education is significantly less than that for a Master's degree or PhD. At the time our survey was open, the national recruitment score sheet for the forthcoming year (2022) had been published, and the points awarded for teaching/education experience and publications/research had been rebalanced (Table 2). No longer were points available for being enrolled on, or completing a PgCert in medical education, and this change is reflected in the free-text comments of some of the survey participants (Box 2), lamenting their choice to enrol on a postgraduate education course which no longer gained any credit in specialist training application criteria scoring. Some university-based posts designed to foster future clinical academics use national recruitment into StR for benchmarking suitable applicants but may also ask for additional attainments, such as possession of a recognised teaching qualification. This is scored locally and does not form part of StR national recruitment application scoring. The National Institute for Health and Care Research has details on integrated academic training for academic clinical fellowships and academic clinical lecturer posts.^16^

**Table 2:** Scoring matrix for application into dental speciality training

Speciality registrar national recruitment scoring matrix			
**2021**			
**Teaching and learning**		**Publications/higher degree**	
Enrolled on teaching qualification	2 points	Peer-reviewed publications published/accepted - one	2 points
Completed teaching qualification	3 points	Publications published/accepted - two or more	4 points
		Possession of Master's degree	2 points
		Possession of PhD	3 points
**2022**			
**Experience in formal teaching events (for example, small group tutorial/lecture)**		**Research/publications**	
Occasionally engaged in teaching (1-5 episodes)	1 point	Letter/book review/non-peer reviewed article/or non-peer reviewed e-publication	1 point
Regularly engaged in teaching (6-10 episodes)	2 points	Case report	3 points
Regularly engaged in teaching (>11 episodes)	4 points	One peer-reviewed publication	4 points
		More than one peer-reviewed publication	5 points

### Similar studies

Our findings regarding interest in medical education were similar to those in a 2018 study by Sethi,^17^who found that the intrinsic desire to improve educational understanding and competency was the most common reason for healthcare professionals to enrol in a medical education qualification. In the same study, Sethi and colleagues also identified similar extrinsic motivations, including continuing a career pathway in medical education. Sethi and colleagues also noted that those participants who initially enrolled for extrinsic reasons gradually developed an interest in education and many continued from certificate to diploma to Masters' programmes. In a study by Lake of a group of medical general medical practitioners (GPs) who were becoming GP trainers and were mandated to complete a medical education course, the authors highlighted the absence of working time dedicated for educational development and that GP trainers struggled to complete a PGCert alongside working.^18^ Similarly, Sethi in 2017 found that clinicians struggled to make time to continue their educational development after completing postgraduate education qualifications.^19^ In reviewing the motivations for enrolling on a PgCert in medical education, our cohort were keen on widening their knowledge and understanding of education and assessment, and a 2022 study by Aitken and colleagues also found that participants on a postgraduate course developed a greater repertoire of teaching skills, methods and theories in medical education.^20^

### Limitations of the study

The response rate of 46% means that we have the views of less than half of the possible pool of 117 participants. We feel it is likely that those who had been involved in a PgCert in medical education would be more motivated to take part in the survey and share their experiences whereas those who had not enrolled on such a course would have little to contribute and would ignore the invitation.

The response rate of 46% is reasonable for an online survey and we believe the results can be generalised to the wider population of DCTs/SHOs and StRs and their experience of enrolling on a PgCert in medical education. Nulty compared online and paper-based surveys, noting response rates of between 20-47% for online surveys,^21^ while Archer^22^examined response rates from online surveys of different types and said that ‘impact evaluation' surveys (the survey type of our study) produced a mean response rate of 51.4%, slightly higher than the response rate for our survey. In an attempt to improve the response rate, we sent reminder emails at weeks two and three with the survey link, which was open for four weeks. Reminder emails have been shown to improve response rates.^23^

We are also assuming that the findings from a group of current and former Cardiff Dental Hospital DCTs/SHOs and StRs are applicable to the wider group of UK junior dental trainees, but also that this is a reasonable assumption.

## Conclusions

This study of early-years dental trainees shows that one of the principal motivations for paying for and finding the time to undertake a postgraduate programme in medical education was to enhance their applications to enter speciality training programmes. The study group felt that the medical education courses had increased their knowledge and understanding of delivery and assessment in medical education, but probably due to their lack of access to opportunities to shape and deliver educational programmes, their preparedness to use their new knowledge was limited. They also noted that because of changes to the scoring of self-assessment applications for recruitment into national speciality training from 2022 onwards, for some of them who were enrolled but had not completed courses, their motivation was somewhat diminished. The enthusiasm of current 2023 DCTs/SHOs to enrol on postgraduate medical education courses will likely diminish and market forces may result in the discontinuation of some of the currently available programmes. Nonetheless, there is now a cohort of junior trainees who are knowledgeable in dental and medical education and may be able to use this experience to benefit future students and trainees.
